# Expressive writing for high-risk drug dependent patients in a primary care clinic: A pilot study

**DOI:** 10.1186/1477-7517-3-34

**Published:** 2006-11-19

**Authors:** Karen A Baikie, Kay Wilhelm, Beverley Johnson, Mary Boskovic, Lucinda Wedgwood, Adam Finch, Gail Huon

**Affiliations:** 1 School of Psychiatry, Black Dog Institute, University of New South Wales, Sydney, NSW, Australia; 2 School of Psychology, University of New South Wales, Sydney, NSW, Australia; 3 The Graduate Research School, The Australian National University, Canberra, ACT, Australia

## Abstract

**Background:**

Previous research has shown that expressive writing is beneficial in terms of both physical and emotional health outcomes. This study aimed to investigate the effectiveness and acceptability of a brief expressive writing intervention for high-risk drug dependent patients in a primary care clinic, and to determine the relationship between linguistic features of writing and health outcomes.

**Methods:**

Participants completed four 15-minute expressive writing tasks over a week, in which they described their thoughts and feelings about a recent stressful event. Self-report measures of physical (SF-12) and psychological health (DASS-21) were administered at baseline and at a two-week follow-up. Fifty-three participants were recruited and 14 (26%) completed all measures.

**Results:**

No statistically significant benefits in physical or psychological health were found, although all outcomes changed in the direction of improvement. The intervention was well-received and was rated as beneficial by participants. The use of more positive emotion words in writing was associated with improvements in depression and stress, and flexibility in first person pronoun use was associated with improvements in anxiety. Increasing use of cognitive process words was associated with worsening depressive mood.

**Conclusion:**

Although no significant benefits in physical and psychological health were found, improvements in psychological wellbeing were associated with certain writing styles and expressive writing was deemed acceptable by high-risk drug dependent patients. Given the difficulties in implementing psychosocial interventions in this population, further research using a larger sample is warranted.

## Background

Randomised controlled trials have demonstrated positive physical and/or psychological benefits of expressive writing [1]. In the expressive writing paradigm, participants write about traumatic, stressful or emotional events in their lives for 3–5 sessions of 15–20 minutes each, over consecutive days or few weeks. Control groups write for the same time about emotionally neutral topics. Early studies with nonclinical groups (e.g., college students) found benefits in objective health outcomes (e.g., doctor visits and grade point average) and some benefits in self-reported physical and psychological health outcomes (e.g., physical symptoms and mood). Lately, researchers have investigated expressive writing as an intervention for clinical groups with physical and/or psychological health problems [2,3]. Meta-analyses have shown significant improvements in health outcomes for both non-clinical [4] and clinical populations [5]. Despite numerous attempts to elucidate an underlying mechanism, to date no single theory can account for the benefits of expressive writing [2]. Indeed, it seems that the benefits probably reflect a host of cognitive, emotional, social and biological processes [6]. However, there is evidence that the development of a coherent narrative, which may help to reorganise and structure traumatic memories, is an important contributor [2].

Because the expressive writing paradigm is a brief, easily administered intervention with potential health benefits, this pilot study examined its acceptability and effectiveness in a population of opioid dependent patients, as they constitute a stigmatised group in whom treatment noncompliance and poor retention rates hinder the delivery of proven psychosocial treatments [7]. Since it is commonly thought that patients with substance use disorders tend to use substances to avoid thoughts and feelings about emotional issues, writing about such experiences may force them to directly confront their emotions and organise their thoughts in ways they may never have done [6]. To provide some context, dependence on drugs such as opioids is a complex, chronic disorder with high comorbidity that requires ongoing multidisciplinary management and the need for effective psychological interventions [8]. In Australia it is estimated that 3.2 percent of males and 1.3 percent of females, aged 18 years and over, meet criteria for a drug use disorder [9]. Treatment is complicated by high rates of co-existing disorders such as depressive disorders, generalised anxiety, phobias, and personality disorders requiring simultaneous management [10], by complex medical needs [11], and by issues such as homelessness, disrupted living conditions, and social isolation [12]. Methadone maintenance programs are efficacious [8], but psychosocial interventions aiming to improve individuals' medical and social functioning are particularly important for those responding less well to methadone maintenance programs. Interventions that are brief, effective, and acceptable to individuals are important both for harm reduction and to enhance the effectiveness of their stabilisation treatment [13].

Expressive writing studies have also explored the relationship between how participants write and observed health benefits using computerised text analysis software [14], to determine how and for whom expressive writing is beneficial. Pennebaker [1,15] suggested that improved health is associated with a linguistic "fingerprint" involving higher use of positive emotion words (e.g., happy, laugh), moderate use of negative emotion words (e.g., sad, angry), and an increasing number of cognitive process words (insight words (e.g., understand, realise) and causal words (e.g., because, reason)) over writing sessions. Several subsequent studies support this relationship for positive emotion words [15,16], although others do not [17-20]. Health benefits were associated with moderate use of negative emotion words in one study [15], higher use in another [17], but unrelated in others [16,18-21]. Increasing use of cognitive words is associated with improved physical health in many [15-17,22,23] but not all studies [18,20,21]. Overall, the findings for positive and negative emotion words are somewhat inconsistent, and increasing cognitive word use seems more consistently related to physical health benefits in healthy samples compared to clinical samples. More recent findings suggest that variation in pronoun use (e.g., I, my) over the course of writing is strongly linked to health improvements [24], perhaps reflecting flexibility in the way people think about interrelationships among themselves, others and events.

This study explored the effectiveness and acceptability of expressive writing in high-risk drug dependent patients in a primary care clinic, in terms of psychological health (depression, anxiety and stress) and physical health (perceived physical and mental disability). The study also investigated the relationship between linguistic markers and health outcomes.

## Methods

### Participants

Participants were recruited from a comprehensive medical, counselling and social welfare service providing methadone access and needle syringe exchange for at-risk youth, sex workers and injecting drug users with a street-based lifestyle, in Kings Cross, Sydney, Australia. To be eligible, participants needed to be stabilised on a treatment program, not in immediate crisis or initial assessment, and aged between 18 and 60 years. No exclusion criteria related to type of substance use were applied, given the wide variety of substances used in this population and the complexity of collecting such data in this small study.

### Procedure

The researchers presented an education session to counsellors at the centre, providing information about expressive writing and inclusion criteria for participation. Counsellors were told not to directly ask participants about their writing, but participants could seek assistance from counsellors if they became distressed. Individuals were informed about the 'diary writing study', investigating the relationship between diary writing and well-being, by staff or via poster at the centre. Interested participants selected a coloured dairy and pen to use and keep at the conclusion of the study. Due to space constraints, researchers administered questionnaires and writing tasks either in the centre's common room, at a quiet café nearby with a free coffee, or participants could elect to write at home. Once informed consent was obtained, participants completed demographic information, baseline measures of physical and psychological health and their first 15-minute expressive writing task. The following instructions for the expressive writing task, adapted from Pennebaker [1] were read to participants and pasted into the front cover of the diaries.

During today's writing session, your task is to write about your very deepest thoughts and feelings about a recent stressful event that has happened to you. It could be something you are experiencing right now or experienced not too long ago. I would like you to write about a topic that is personally relevant to you. In your writing, the most important thing is that you really let go and explore your very deepest emotions and thoughts related to this event. You may write about how this experience has affected your view of yourself, others, or of the world in general. You might tie your topic to your relationships with others, including parents, lovers, family, or relatives, or who you are in general as a person. The only rule about the writing task is that you are to write continuously, without stopping, for about 15 minutes or until you are unable to write anymore. Do not worry about spelling, grammar, or sentence structure. All of your writing will be completely confidential and anonymous. It is important for you to know that your name will not be connected in any way with your writing. Over the four days of diary writing, you may write about the same experience or event on each day or you may write about different stressful events.

Those writing on site returned to complete three additional writing tasks on separate days over the following week or so. Diaries were kept in a secure location. Those writing at home were told to write for 15 minutes on four separate days and bring their diaries back one week later. Approximately two weeks after recruitment, participants completed measures of physical and psychological health and acceptability of expressive writing, and were given a certificate of participation. Data was collected between June and August 2004. Two months later a feedback session was provided to inform participants about study outcomes. Participants provided anecdotal evidence of the acceptability and utility of the writing task at this session. The study was approved by the Human Research Committee of the South Eastern Sydney Area Health Service – Eastern Section and by the University of New South Wales Human Ethics Research Committee.

### Measures

#### Psychological Health

The *Depression Anxiety Stress Scales-21 *[DASS-21; [25]] is a self-report scale measuring negative emotional states. The Depression subscale assesses dysphoria, hopelessness, and loss of self-esteem and incentive, the Anxiety subscale assesses autonomic arousal and fearfulness, and the Stress subscale assesses tension, irritability, and being easily upset or agitated [25,26]. Each subscale has 7 items and participants rate the extent to which they experienced each state over the past week on a 4-point scale ranging from 0 (*did not apply to me at all*) to 4 (*applied to me very much or most of the time*) with higher scores indicating greater psychological distress. The DASS-12 has very good internal consistency, a clean factor structure, acceptable test-retest stability, and good construct validity in both clinical and nonclinical samples [27].

#### Physical Health

The *12-item Short Form Health Survey *[SF-12; [28]] is a self-report summary measure of perceived physical and mental disability. The scale has a question about perceived general health, eight questions assessing the extent to which current health limits physical activities, vitality and social functioning, and three questions assessing emotional distress. It provides a Mental Component Summary (MCS) score and a Physical Component Summary (PCS) score, with lower scores indicating greater disability. The SF-12 has satisfactory psychometric properties and is reliable and valid in a variety of clinical populations (Ware et al., 1996). The SF-12 instructions were modified to assess health over the past one week (rather than to four weeks) to correspond with the study design.

#### Acceptability of Writing questionnaire

Participants rated the extent to which the writing sessions were beneficial or helpful on a 7-point scale from 1 (*not at all*) to 7 (*a great deal*). They also indicated how often they had written in their diary since the study and whether they would participate again if they had their time over (*yes/no*).

#### Linguistic Analysis

*Linguistic Inquiry and Word Count *[LIWC; [14]] is a computerized text analysis system which analyses written text on a word-by-word basis and determines the percentage of words that are assigned to up to 82 pre-defined language categories using a dictionary of 2300 words and word stems. LIWC analysis has demonstrated good internal consistency across different writing samples and topics [29], and external validity is demonstrated by high correlations between independent judges' ratings of written text and the LIWC output [16]. People's word usage patterns measured by LIWC2001 satisfy the basic psychometric requirements of stability over time and consistency across context [30]. Whilst LIWC, like any other text analysis program, cannot take into consideration context, syntax, linguistic devices such as irony and sarcasm, and the problem of multiple meanings of words [29,31], it is able to provide an objective, rapid analysis of diverse text samples, making it a valuable tool for expressive writing research [30,32].

Based on previous studies, four LIWC variables were included for analysis. Mean scores over four writing sessions were calculated for positive emotion words (e.g., happy, pretty, love, win) and negative emotion words (e.g., anger, grief, guilt, ugly). Causal (e.g., because, why, reason) and insight (e.g., realise, see, understand) word scores for each day were standardized and summed, and a cognitive change score from first to last day of writing was obtained using the following algorithm: (Day 4 × 3) + (Day 3 × 1) - (Day 2 × 1) - (Day 1 × 3), with higher scores indicating an increase in cognitive words over the course of writing [15]. For the one participant with only 3 writing tasks, cognitive change score was obtained using (Day 4) - (Day 1) as done in other studies [16]. Flexibility in pronoun usage was calculated as the mean of |Day 1-Day 2|, |Day 2-Day 3|, |Day 3-Day 4| for first person pronoun words (e.g., I, me, my) [33].

## Results

### Participant Characteristics

Fifty three participants (25 females, mean age 34.1 range 20–54) were recruited. Three participants did not complete all baseline questionnaires and 13 took the diary but did not return. Twelve completed only 1 writing task, 4 completed 2 tasks, 3 completed 3 tasks, and 18 completed all 4 tasks. Of the original 53, 14 (26%) completed 3 or 4 diary tasks and the follow-up measures (7 females, mean age 31.8 range 23–48).

Table 1 shows demographic and health characteristics of 48 participants providing complete data at baseline and 14 participants completing follow-up data. There were no significant differences between completers and dropouts in age, gender, education level, living arrangement, accommodation status, baseline depression, anxiety, stress or general health status (all *p*'s > .05), suggesting that sample attrition was unrelated to demographics, psychological or physical health. Reference to normative data reveals that the sample means fall within the severe, extremely severe, and moderate levels for depression, anxiety and stress respectively [25] and within the moderate and mild disability range for mental and physical health status respectively [28]. On average, this sample reported higher levels of psychological problems and, to a lesser extent, physical health problems, than the general population.

**Table 1:** Demographic and psychological/physical health characteristics of participants who completed baseline measures (N = 48) and follow-up measures (N = 14).

	Baseline sample (N = 48)		Follow-up sample (N = 14)	
Characteristic	n	%	n	%

Gender				
Females	23	47.9	7	50.0
Males	25	52.1	7	50.0

Education Level				
Finished school and did university/college/trade course	7	14.6	0	0.0
Left school early and did college/trade course	22	45.8	7	50.0
Left school after 10–11 yrs and no course	7	14.6	5	35.7
Less than 10 yrs school and no course	12	25.0	2	14.3

Living arrangement				
Living alone	21	43.8	6	42.9
Living with partner	16	33.3	6	42.9
Living with partner and children	3	6.3	0	0.0
Living with friend/s or family	8	16.7	2	14.3

Accommodation status				
Live in own house or flat	19	39.6	5	35.7
Live in parents' home	3	6.3	0	0.0
Live in boarding house or hostel	12	25.0	5	35.7
No fixed address or homeless	13	27.1	4	28.6
Other	1	2.1	0	0.0

Characteristic	Mean	SD	Mean	SD

Age	34.3	8.8	31.8	6.7
DASS-21 Depression score	23.3	11.4	25.4	9.0
DASS-21 Anxiety score	20.1	10.4	22.0	8.6
DASS-21 Stress score	24.6	10.0	25.0	7.3
SF-12 Mental Component score	34.8	11.2	32.3	9.8
SF-12 Physical Component score	41.2	9.2	39.7	7.9

### Writing Topics

Many participants wrote about more than one topic during any single writing task. Of the final 55 writing tasks, 19 (34%) concerned problems in family relationships, 10 (19%) dealt with problems in other relationships, 13 (23%) involved legal difficulties, 13 (23%) discussed concern about physical or psychological health, 11 (20%) mentioned housing and homelessness, 8 (14%) concerned isolation and identity, 8 (14%) involved violence and assault, 4 (7%) mentioned financial problems, and 6 (10%) concerned general daily matters.

### Physical and Psychological Health Outcomes

Repeated measures ANOVAs were conducted on each of the subscales of the DASS-21 and SF-12 to determine the effect of expressive writing on health at two-week follow-up. Group means for each subscale changed in the direction of improved health, although the changes failed to reach statistical significance (see Table 2). Calculated effect sizes (partial η^2^) and observed power calculations indicate that a larger sample size would be required to reach a .80 power convention for an adequate test of outcome effects.

**Table 2:** Mean and standard deviations for DASS-21 and SF-12 subscales at baseline and two-week follow-up (*n *= 14).

Measure	Baseline		Follow-Up		*F*	p	partial	Observed
					
	Mean	SD	Mean	SD	(1,13)		η^2^	power
DASS-21 Depression	25.4	9.0	21.0	9.9	2.95	0.11	.19	.36
DASS-21 Anxiety	22.0	8.6	17.0	11.3	3.61	0.08	.22	.42
DASS-21 Stress	25.0	7.3	21.3	9.1	2.17	0.17	.14	.28
SF-12 Physical	39.7	7.9	41.2	12.5	0.26	0.62	.02	.08
SF-12 Mental	32.3	9.8	35.2	11.3	2.28	0.15	.15	.29

### Relationship between LIWC Indices and Health Outcomes

Relationships between health outcomes and the use of positive emotion words, negative emotion words, increase in cognitive process words and flexibility in first person pronouns were investigated. Bivariate correlations between these four LIWC indices and pre to post change on the five health outcomes are shown in Table 3. Use of more positive emotion words was significantly associated with improvements in depression and stress. Contrary to expectation, increase in use of cognitive process words from first to last writing was associated with a worsening in depression scores. Flexibility in use of first person pronouns was significantly associated with improvements in anxiety.

**Table 3:** Correlations between LIWC indices and change in psychological and physical health outcomes.

LIWC Index	Change in Psychological and Physical Health^a^				
	
	DASS Depression	DASS Anxiety	DASS Stress	SF-12 Mental	SF-12 Physical
Mean Positive Emotion Words	-.608*	-.532	-.721**	.242	.290
Mean Negative Emotion Words	.223	.013	.407	-.196	.034
Change in Cognitive Words	.589*	.239	.194	-.301	.303
Flexibility in First Person Pronouns	-.425	-.778**	-.481	.062	.175

^a ^Change scores calculated as post-score minus pre-score. For DASS subscales, negative change scores indicate improvement, whereas for SF-12 subscales positive change scores indicate improvement.

* p < 0.05

** p < 0.005

### Acceptability of Diary Writing

Diary writing was rated as moderately to extremely beneficial/useful (score ≥ 4 on a 7-point scale) by 12 (86%) of 14 participants. Five (36%) participants continued to write in their diary after the study and 11 (79%) said they would participate in the study if they had their time over again. Anecdotal feedback to researchers during the study and at the follow-up information session indicated that many participants were enthusiastic about expressive writing and felt that it was helpful.

## Discussion

This preliminary investigation of expressive writing in a drug dependent population found no statistically significant benefits in self-reported physical and psychological health at two-week follow-up, although all outcome measures changed in the direction of improvement. Given the high-risk population, it is noteworthy that participants' health did not get worse following the intervention. There are several possible methodological explanations for the non-significant effects.

First, and most obviously, the power of the study to detect significance was limited by the small final sample size, due to high attrition (74%). Expressive writing studies usually assess health outcomes after a month or longer, however the transient nature of this population necessitated a shorter follow-up period because of the likelihood of even greater attrition. Significant health improvements may have been detectable over a longer timeframe or with a larger sample. Also, availability of resources prevented the inclusion of a control group, which would have enabled a comparison between outcomes for expressive writing and neutral writing.

The intervention's impact may have been limited by the instruction to write about a "recent stressful event", in contrast to "the most traumatic and upsetting experiences of their entire lives" generally used in expressive writing studies [1]. The high-risk nature of this population prompted a more cautious approach to minimise the possibility of adverse consequences, however, participants may have written about less emotionally salient events than in other studies, limiting the potential for health benefits. Although participants were instructed to "really let go and explore their very deepest emotions and thoughts", examination of writing tasks showed that some participants wrote about stressful events in a more descriptive manner rather than writing about their feelings or thoughts. Some participants may have avoided the more in-depth emotional and cognitive processing regarded as a critical element underlying the benefits of expressive writing [6].

Participants' preexisting health status was significantly poorer than both the general population [28] and participants in non-clinical expressive writing studies [4]. The improvements in immune functioning thought to relate to improved health after writing [34] may have had limited short-term impact in a population with poorer health and chronic conditions. In addition, it is possible that participants who simultaneously use a number of substances may have poorer outcomes, but detailed assessment of poly-substance use was beyond the scope of this study.

Finally, the availability of resources prevented access to objective measures of health and limited the number and complexity of self-report measures that could be administered. Significant benefits may have been observable on objective or other self-report measures more specific to the health problems common to this population.

We found that using more positive emotion words was significantly associated with improvements in depression and stress. This finding extends previous research linking increased use of positive emotions with improvements in physical health [15,16]. Thinking positively is generally beneficial in improving psychological functioning [35] and optimistic individuals show better psychological adjustment [36]. Flexibility in use of first person pronouns was significantly associated with improvements in anxiety, which complements previous research showing an association between variation in pronoun use and physical health benefits [24]. Unexpectedly, increasing number of cognitive process words from first to last writing was associated with worsening depression scores. Whilst increasing cognitive word use generally predicts improved physical health, other studies have also found it to be unrelated [21] or negatively related to psychological health [18]. In this sample, increasing use of cognitive words may reflect the start of a process of dealing with emotional material that had previously been 'kept at bay' by drug use. No linguistic markers were related to SF-12 physical or mental health outcomes. The linguistic predictors of physical and psychological health are likely to be different and further exploration of these relationships in different populations is warranted.

It is nevertheless noteworthy that in a population that generally has difficulty accessing psychological interventions, expressive writing was well-received and regarded as useful by the participants themselves. Researchers and counsellors were surprised by the degree of enthusiasm with which diary writing was received, with many participants continuing to write in their diaries after the study.

## Conclusion

In this preliminary study of high-risk drug dependent patients in a primary care clinic, a brief expressive writing intervention was acceptable and well-received by participants. Although no statistically significant improvements in self-reported physical or psychological health outcomes were found, participants' health did not worsen and linguistic analysis demonstrated some significant relationships. Given the difficulty implementing psychosocial interventions in this population and previous findings of health benefits after expressive writing, further research with a larger sample is warranted, as expressive writing may prove to be a useful intervention for harm reduction in people with substance abuse problems.

## Competing interests

The author(s) declare that they have no competing interests.

## Authors' contributions

KB contributed to study design, coordination and supervision, performed data analysis and interpretation, and drafted the manuscript. KW conceived of the study, participated in its design and coordination, and helped to draft the manuscript. BJ contributed to study coordination and statistical analysis. MB, LW and AF participated in study planning, coordination and data collection. GH participated in study planning and supervision of BJ and MB. All authors read and approved the final manuscript.
